# Genome-wide investigation and expression analysis suggest diverse roles and genetic redundancy of Pht1 family genes in response to Pi deficiency in tomato

**DOI:** 10.1186/1471-2229-14-61

**Published:** 2014-03-11

**Authors:** Aiqun Chen, Xiao Chen, Huimin Wang, Dehua Liao, Mian Gu, Hongye Qu, Shubin Sun, Guohua Xu

**Affiliations:** 1 State Key Laboratory of Crop Genetics and Germplasm Enhancement, College of Resources and Environmental Sciences, Nanjing Agricultural University, Nanjing 210095, China

**Keywords:** Phosphate transporter, Pht1 family, Evolution, Functional divergence, Expression pattern, *Solanum lycopersicum*

## Abstract

**Background:**

Phosphorus (P) deficiency is one of the major nutrient stresses limiting plant growth. The uptake of P by plants is well considered to be mediated by a number of high-affinity phosphate (Pi) transporters belonging to the Pht1 family. Although the Pht1 genes have been extensively identified in several plant species, there is a lack of systematic analysis of the Pht1 gene family in any solanaceous species thus far.

**Results:**

Here, we report the genome-wide analysis, phylogenetic evolution and expression patterns of the Pht1 genes in tomato (*Solanum lycopersicum*). A total of eight putative Pht1 genes (*LePT1* to *8*), distributed on three chromosomes (3, 6 and 9), were identified through extensive searches of the released tomato genome sequence database. Chromosomal organization and phylogenetic tree analysis suggested that the six Pht1 paralogues, *LePT1*/3, *LePT2*/6 and *LePT4*/5, which were assigned into three pairs with very close physical distance, were produced from recent tandem duplication events that occurred after Solanaceae splitting with other dicot families. Expression analysis of these Pht1 members revealed that except *LePT8*, of which the transcript was undetectable in all tissues, the other seven paralogues showed differential but partial-overlapping expression patterns. *LePT1* and *LePT7* were ubiquitously expressed in all tissues examined, and their transcripts were induced abundantly in response to Pi starvation; *LePT2* and *LePT6*, the two paralogues harboring identical coding sequence, were predominantly expressed in Pi-deficient roots; *LePT3*, *LePT4* and *LePT5* were strongly activated in the roots colonized by arbuscular mycorrhizal fungi under low-P, but not high-P condition. Histochemical analysis revealed that a 1250-bp *LePT3* promoter fragment and a 471-bp *LePT5* promoter fragment containing the two elements, MYCS and P1BS, were sufficient to direct the GUS reporter expression in mycorrhizal roots and were limited to distinct cells harboring AM fungal structures. Additionally, the four paralogues, *LePT1*, *LePT2*, *LePT6* and *LePT7*, were very significantly down-regulated in the mycorrhizal roots under low Pi supply condition.

**Conclusions:**

The results obtained from this study provide new insights into the evolutionary expansion, functional divergence and genetic redundancy of the Pht1 genes in response to Pi deficiency and mycorrhizal symbiosis in tomato.

## Background

Phosphorus (P) is one of the three most essential macronutrients required by plants. It is well recognized as serving a wide range of structural and biological roles, such as energy metabolism, signal transduction, biosynthesis of macromolecules, modulation of respiration, photosynthesis and other metabolic processes [1]. The primary source for P uptake by plants is orthophosphate (Pi) in soil. Due to the slow diffusion rate and chemical fixation, P is widely considered to be one of the most difficult nutrients for plants to forage, and often a major limiting factor to crop yields [2,3].

The Pi concentration in soil solution is commonly no more than 10 μM, whereas plant cells need to maintain their cytoplasmic Pi concentrations at a millimolar range [4,5], which determines the requirement of metabolic energy and specific transport systems for plants to acquire Pi from soils [6,7]. In the past decades, aided by systematic studies of molecular biology and functional genomics of model plants, a great deal of knowledge about the mechanisms of Pi transport by plants has been accumulated, revealing that the uptake and subsequently redistribution of Pi within plants are mediated by a number of phosphate (Pi) transporters with different affinities that located in the plasma or organelle membranes [8].

The first gene encoding plant Pi transporter (AtPT1) was isolated from *Arabidopsis*[9] and showed high sequence identity to the genes encoding high-affinity Pi transporters in *Saccharomyces cerevisiae* (Pho84) [10] and in *Glomus versiforme* (GvPT) [11]. The later studies further led to the isolation of other eight homologues exhibiting substantial identities to the *AtPT1* in the *Arabidopsis* genome [12], suggesting the expansion of Pi transporter genes in higher plants during evolution. By now, with the completion of whole genome analysis of model plants, such as *Arabidopsis* and rice, dozens of homologous genes encoding different affinities and groups of Pi transporters have been identified in various plant species by comparative genomic approaches [13]. Studies on the protein sequences and phylogenetic relatedness revealed that most of the Pi transporters identified so far are typical of H^+^/Pi symporters, and could be grouped into the high-affinity Pht1 family included in the super facilitator superfamily (MFS) [14-16].

Earlier studies on the regulation and tissue/cellular distribution indicated that members of the Pht1 family in many species are divergent in function and differentially expressed during plant development or in response to different P status [17,18]. The relatively high levels of transcripts or proteins of some Pht1 genes in roots, especially in root epidermis and root hairs, in response to Pi deficiency well support a role of these genes in Pi capture and uptake [19,20]. For example, in *Arabidopsis*, eight of the nine Pht1 genes were expressed in roots and two members, *AtPT1* and *AtPT4*, had the highest expression levels in response to Pi deficiency. Knock out of either of the two genes showed significant defects in P uptake under a low Pi supply condition [21,22]. In some cases, the transcripts of some Pht1 members are more widely distributed throughout plant tissues and showed less responses to Pi deficiency, providing strong evidence to support that some of the Pht1 members may be implicated in the internal mobilization of Pi, such as loading or unloading from the xylem or phloem and deposition into seeds or other storage organs [19,23-25]. In addition to the Pi-responsive Pht1 genes, an increasing number of arbuscular mycorrhiza-induced Pi transporters belonging to the Pht1 family have been identified from several plant families, and their functions have been repeatedly documented to be associated with Pi uptake at the intraradical symbiotic interface [26-33].

Tomato, a member of the Solanaceae, is not only a world-wide major vegetable crop plant, but also a model plant for biological and genetic researches based on its relatively low-copy DNA sequence and the nearly complete genome sequencing [34]. Although previous studies have characterized the potential roles of a few individual Pht1 members in tomato [30,35], there is a lack of genome-wide analysis of the Pht1 gene family in tomato and also in any other solanaceous species thus far. Moreover, compared to the other model species, such as *Arabidopsis* from Brassicaceae and rice from Gramineae, the evolutionary mechanisms, transcriptional regulation and possible functions of solanaceous Pht1 genes in Pi acquisition and mobilization still needs to be well explored [36-39].

In the current work, we reported the genome-wide identification and comparative characterization of Pht1 family genes in tomato and potato, and further investigated the expression patterns of tomato Pht1 genes in response to AM fungi inoculation under low- and high-P supply condition. The analysis in this study mainly focused on the chromosomal organization, phylogenetic evolution, tissue-specific expression and regulation of each member of the tomato Pht1 family. The results obtained from this study would not only strengthen our understanding on the molecular mechanisms underlying the evolutionary expansion, conservation and functional divergence of the Pht1 genes in tomato, but also provide valuable clues for the further comparative genomic studies across the whole Solanaceae family.

## Results

### Identification of Pht1 family genes in tomato

Previously, five Pht1 genes (three with full-length and two with partial mRNA sequence) encoding for putative high-affinity Phosphate (Pi) transporters (PT) in tomato have been reported [30,40]. In order to determine whether there are any further members, as yet unidentified, comprising the tomato Pht1 family, the mRNA and amino acid sequences of *Arabidopsis* and rice Pht1 genes were employed for BLASTN and TBLASTN searches against the recently released tomato genomic sequence database (http://solgenomics.net/), which resulted in the identification of a total of eight non-allelic sequences as the putative tomato Pht1 genes (Additional file 1). BLAST searches of these sequences against the NCBI database demonstrated that five of the eight sequences were identical to the accessioned tomato Pht1 genes, *LePT1* to *5*. The rest three putative genes (named as *LePT6* to *8*), which were newly identified in this study, showed high levels of sequence identity to the known Pht1 genes from tomato and other plant species (Table 1). Moreover, *LePT6*, which represents a distinct locus, harbors its coding sequence identical to that of the known *LePT2*, but with much difference in un-translated regions between the two homologues (Additional file 2).

**Table 1:** Identity matrix for the eight putative tomato Pht1 genes

		**Amino acid identity (%)**							
		**LePT1**	**LePT2**	**LePT3**	**LePT4**	**LePT5**	**LePT6**	**LePT7**	**LePT8**
Nucleotide Identity (%)	LePT1	-	80.7	85.3	61.3	62.1	80.7	82.5	81.1
	LePT2	71.0	-	77.2	60.5	60.1	100.0	73.9	73.1
	LePT3	76.1	70.4	-	60.5	61.5	77.2	77.0	76.0
	LePT4	61.2	59.3	59.2	-	89.8	60.5	59.3	58.0
	LePT5	60.9	57.6	59.4	86.0	-	60.1	59.3	58.7
	LePT6	71.0	100.0	70.4	59.3	57.6	-	73.9	73.1
	LePT7	74.6	67.1	70.5	59.0	59.9	67.1	-	92.5
	LePT8	75.2	67.0	69.5	58.1	59.0	67.0	91.4	-

**Figure 1:**
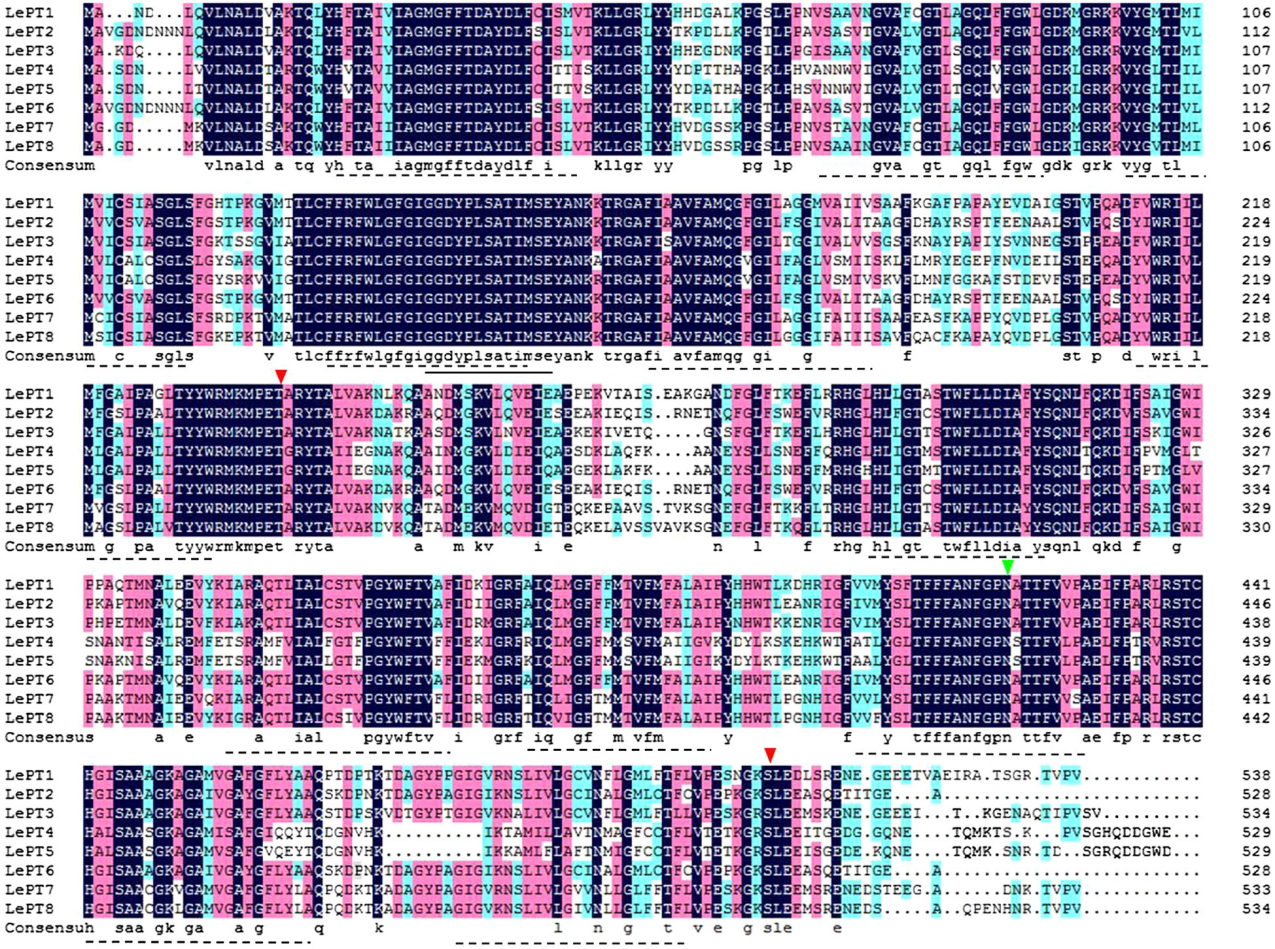
**Predicted amino acid sequences of the eight tomato Pht1 genes *LePT1 *to *8*.** Sequence alignment analysis was carried out using multiple alignment algorithm wrapped within the DNAMAN 7.0 program (http://www.lynnon.com/). Identical amino acids are shaded and gaps are indicated by dots. The consensus sites for phosphorylation by protein kinase C and casein kinase II are shown by two arrowheads with red colour and the conserved N-glycosylation residue is shown by a green arrowhead. The characteristic Pht1 signature was underlined. The transmembrane domains (broken underline) were predicted by the Toppred algorithm (http://bioweb.pasteur.fr/seqanal/interfaces/toppred.html).

Comparative analysis of the full-length deduced polypeptides revealed that the eight Pht1 proteins contain 528-538 amino acids with 12 predicted transmembrane-spinning domains, similar to the molecular feature of Pht1 transporters from other plant species. Additionally, all the tomato Pht1 amino acid sequences share the consensus sites for phosphorylation by protein kinase C and casein kinase II and conserved residue for N-glycosylation (Figure 1). Using the DNAMAN multiple sequence alignment program, the conserved domain, GGDYPLSATIxSE, which have been suggested to be a typical signature of Pht1 proteins, was also identified in all of these proteins (Figure 1). These findings led to the suggestion that all the identified genes could be considered as tomato Pht1 genes.

A further blast searches against the tomato EST database at NCBI, SGN and TIGR database revealed that except *LePT8*, the other two newly identified Pht1 genes, *LePT6* and *LePT7*, could matched perfectly to at least one significant EST sequences, indicating that the two genes, like their previously reported five paralogues, are transcriptionally active in a certain tissues. It should also be emphasized that except the eight Pht1 genes mentioned above, another sequence (named as *LePTx* in this study) identified in the tomato scaffold database searches also showed very high identity to the tomato *LePT7* and *LePT8* genes, but may be inactive due to the inclusion of some nonsense mutations and indels (insertions and deletions) within its putative coding region (Additional file 3), as well as to the absence of any EST sequence exactly matching.

### Identification of tomato Pht1 homologues in potato and comparative analysis of these genes between the two solanaceous genomes

As a near complete set of potato genome sequences were also recently available at the SGN database [41], for further investigating the evolutionary conservation and divergence of Pht1 gene family between the two solanaceous species, the potato genome sequence database were extensively searched using the tomato Pht1 genes as queries, leading to the identification of a total of 10 distinct genes as putative potato Pht1 genes (Additional file 4). Sequence comparison of the potato Pht1 genes revealed similar amino acid sizes and high sequence identities to their corresponding orthologues from tomato (Additional files 4 and 5). It should be noted that there also exist two other sequences in the potato genome that showed substantial homology to the plant Pht1 genes, but may be pseudogenes (named as *StPTx1* and *StPTx2*, respectively), due to the presence of some nonsense mutations and the inclusion of some indels (insertions and deletions) within their putative coding regions.

**Figure 2:**
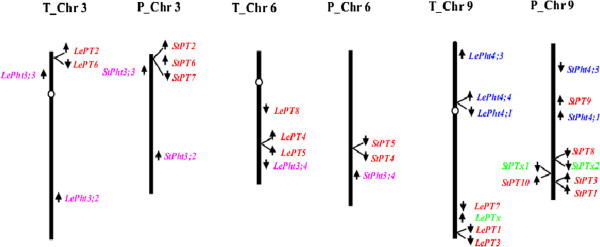
**Distribution of Pht1 genes (*LePT1 *to *8 *and *StPT1 *to 10) on the tomato (T) and potato (P) chromosomes.** Chromosome numbers are shown at the top of each bar. The arrows next to the gene names indicate the direction of transcription. *LePTx*, *StPTx1* and *StPTx2* are putative Pht1 pseudogenes residual in the tomato and potato genomes. The genes from other two families, Pht3 and Pht4, encoding putative Pi transporter or carrier with no homology to the Pht1 proteins, were also labeled on the corresponding chromosomes of the two plants.

Similar to the high sequence identity between the tomato and potato Pht1 members, high conservation of chromosomal organization of the Pht1 homologues from the two solanaceous species could also be observed. Figure 2 shows the localizations of Pht1 genes on the tomato and potato chromosomes. It was revealed that the distribution of the tomato and potato Pht1 genes were obviously uneven, and concentrated on only three (3, 6, and 9) chromosomes of the two plants. In addition, the supposed three pseudogenes (*LePTx*, *StPTx1* and *StPTx2*) were restrictedly assigned on the chromosome 9 of the two plants. Interestingly, except some individual members, such as *LePT8* on tomato chromosome 6 and *StPT9* on potato chromosome 9, most of the other Pht1 genes/pseudogenes on the corresponding chromosomes were distributed in clusters with very short physical distance (Additional file 1, Figure 2), suggesting that these clustered genes may be produced from independent tandem duplications during the evolution of Solanaceae Pht1 gene family.

In addition to the Pht1 gene themselves, the potential genes surrounding each of the Pht1 members were also carefully surveyed, resulting in the identification of several putative genes exhibiting substantial homology to plant Pht3 and Pht4 family genes on the corresponding chromosomes (Figure 2). By comparing the locations of these genes, such as *LePht3;4* and *StPht3;4*, we confirmed the existence of two segmental inversions associated with the long arms of tomato/potato chromosomes 6 and 9, which resulted in the inverted linear order of the orthologous pairs of PT4/PT5 and PT1/PT3 on the corresponding chromosomal regions between the two species (Figure 2).

### Phylogenetic analysis of Pht1 gene family in tomato and other plant species

**Figure 3:**
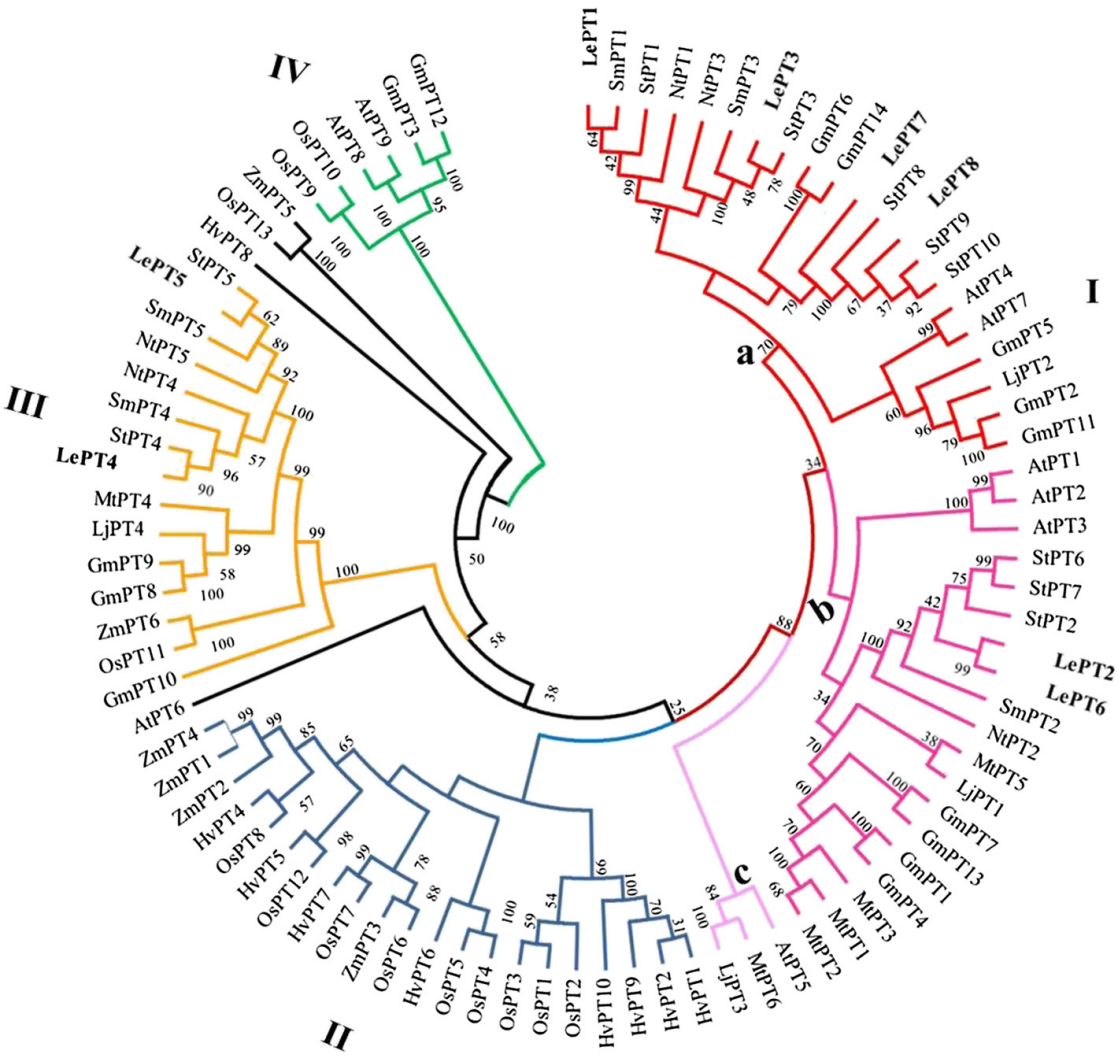
**Phylogenetic analysis of tomato Pht1 genes and other plant Pht1 homologs.** An unrooted phylogenetic tree of the plant Pht1 proteins was constructed using the neighbor-joining method with MEGA 5.0 program. Transporters and corresponding plant species are: tomato, LePT1 to 8 [30,42], this study; potato, StPT1 to 10 [30,43,44], this study; tobacco, NtPT1 to 5 [45,46]; eggplant, SmPT1 to 5 [45,46]; *Arabidopsis thaliana*, AtPT1 to 9 [47]; *Medicago truncatula*, MtPT1 to 6 [8,48,49]; *Lotus japonicus*, LjPT1 to 4 [31,50]; Soybean, GmPT1 to 14 [13]; Rice, OsPT1 to 13 [28]; Barley, HvPT1 to 12 [14,51]; Maize, ZmPT1 to 6 [18,29].

In order to perform a comprehensive analysis of evolutionary relationships among Pht1 genes between tomato and other plant species, including the eight Pht1 proteins of tomato, a total of 90 plant Pht1 protein sequences, representing 11 species from four plant families, Gramineae, Brassicaceae, Leguminosae and Solanaceae, were aligned and used to construct an unrooted phylogenetic tree. As shown in Figure 3, except AtPT6 from *Arabidopsis* and HvPT8, OsPT13 and ZmPT5 from each of the three graminaceous species, barley, rice and maize, the other plant Pht1 proteins in the Neighbour-Joining tree were well clustered into four distinct groups, consisting of one dicot-specific group (I), one monocot-specific group (II) and two mixed groups (III and IV, respectively) with members from both dicots and monocots.

The Group I harbors the proteins exclusively from the dicotyledonous species, in which they are subgrouped by phylogeny, and could be further classified into three subgroups (named as subgroup a, b and c, respectively). In addition, except the subgroup c, which only includes one member from each of the three species, *Arabidopsis*, *Medicago* and *Lotus japonicus*, both of the other two subgroups, a and b, contain multiple Pht1 members from the three plant families, Leguminosae, Brassicaceae and Solanaceae. For tomato, six of the eight Pht1 transporters (LePT1 to 3 and LePT6 to 8) fall into two of the three subgroups. Within subgroup a, the two tomato members, LePT1 and LePT3, group together with their orthologous pairs from potato, eggplant and tobacco, to the exclusion of other two paralogues, LePT7 and LePT8, which group together with other three potato homologues and forms a cluster with two soybean homologues, GmPT6 and GmPT14. Within the dicot subgroup b, the two tomato paralogues, LePT2 and LePT6, group closely, and cluster together with three potato homologues, StPT2, StPT6 and StPT7. The grouping of LePT2 and LePT6 was expected as the two paralogues contain identical coding sequences. The rest two paralogues, *LePT4* and *LePT5*, were found to be assigned only into Group III, although the Group IV also contains the members from both dicots and monocots. Within Group III, the two genes, like their two paralogues *LePT1* and *LePT3* in Group I, also group together with other solanaceous orthologues by forming an independent Solanaceae clade consisting of two subclasses. Both of the subclasses contain the orthologous pairs of PT4 or PT5 from tomato, potato eggplant and tobacco, suggesting that the duplication events associated with the arising of PT4 and PT5, as well as PT1 and PT3 in tomato and other solanaceous species, occurred before the speciation of Solanaceae lineages. Additionally, most of the Pht1 proteins in the Group III, including the PT4 and PT5 orthologous pairs, have been experimentally evidenced to be strongly induced in the roots colonized by arbuscular mycorrhizal (AM) fungi [8]. Moreover, Pht1 members from *Arabidopsis*, of which the roots are unable to form AM symbiosis, are all absent from Group III. Interestingly, although the Group IV contains much fewer members as compared with the other three Groups, there exist two members from each of three species, *Arabidopsis*, rice and soybean, but no homologues from any of the solanaceous species in the Group IV, suggesting that the corresponding orthologues from solanaceous lineages have lost after Solanaceae separation with Brassicaceae and Leguminosae.

### Expression analysis of the tomato Pht1 genes in different tissues under low-P condition

**Figure 4:**
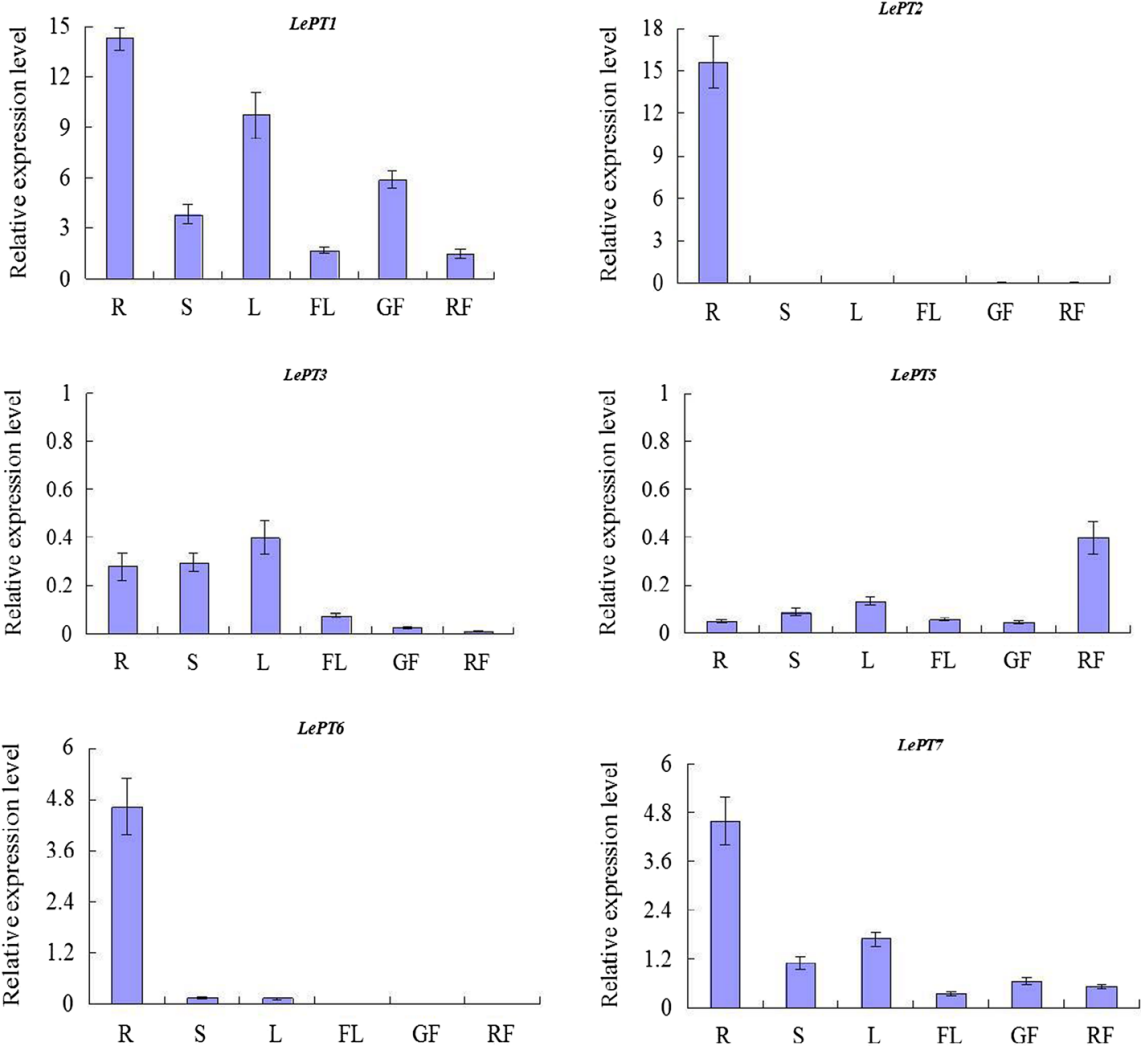
**Tissue-specific expression analysis of tomato Pht1 genes.** The RNA were prepared from different tissues, including roots (R), stems (S), young leaves (L), flowers (FL), as well as fruits at green (GF) and ripe (RF) stages. The relative expression levels of each of the tomato Pht1 genes were indicated as percentage of the constitutive *Actin* expression activity. Each bar was the mean of three biological replications with standard error.

In this study, for gaining better understanding of the possible functions of specific Pht1 gene in tomato, the tissue-specific expression patterns of each tomato Pht1 gene were examined in various tissues, including roots, stems, young leaves, flowers, as well as fruits at young and ripe stages using Real-time RT-PCR. The quantitative data showed that except *LePT4* and *LePT8*, of which the transcripts were not detectable in all tissues examined, the transcripts of other Pht1 paralogues were all detectable in a certain tissues and showed distinct but partially overlapping expression profiles (Figure 4).

*LePT1* was expressed in all tissues examined, and its transcripts were detected abundantly in roots and leaves, and to a lesser extent in stems and flowers, as well as in fruits. The transcripts of *LePT1* in green fruits were four times more than those in ripe fruits. In contrast to the ubiquitous expression profiles of *LePT1*, the expression of *LePT2* showed relatively distinct tissue-specific profiles, with its transcripts intensively in roots and extremely faintly in some of other tissues, such as in green and ripe fruits. The expression patterns of the *LePT3* and *LePT5* were a little similar, as both of the two genes were expressed very weakly in all tissues. Even so, the highest transcript level for *LePT*5 was detected in ripe fruits, and was about ten times more than that in green fruits. *LePT6*, the closest fellow of *LePT2* in phylogeny, was also dominantly expressed in roots, but with only one-third of the expression level of *LePT2* in the root tissues. Additionally, very weak transcript levels of this gene were also detectable in stems and leaves. *LePT7* was also ubiquitously expressed in all tissues, and had a very similar expression tendency, but significant lower expression levels in all tissues as compared to its paralogue, *LePT1* (Figure 4). The differential but overlapping expression of the Pht1 genes well mirrors the evolutionary conservation and functional divergence of Pht1 transporters in tomato plants.

### Expression analysis of tomato Pht1 genes in response to AMF colonization under low and high Pi supply conditions

**Figure 5:**
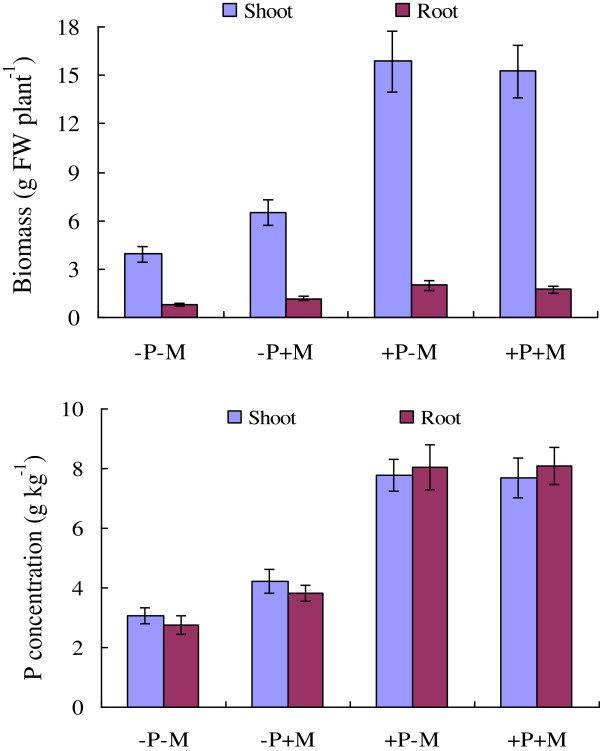
**Effects of mycorrhizal fungal colonization on tomato biomass (fresh weight, FW) and P concentrations under low Pi (0.05 mM) and high Pi (1 mM) supply condition.** –P and + P represent supply of 0.05 mM and 1 mM Pi, respectively; –M and + M represent the inoculation with autoclaved and active inoculum containing arbuscular mycorrhizal fungi *Glomus intraradices* (Gi), respectively. Error bars indicate SE (n = 3).

Since the expression of some Pht1 genes in tomato and also in other plant species, have been characterized to be AM-inducible and Pi-responsive [21,52], the relative expression levels of each tomato Pht1 member were thus further determined in roots and leaves in response to AM Fungi (*Glomus intraradices*) colonization under low (50 μM) and high (1 mM) Pi supply condition. As shown in Figure 5, colonization of AM fungi increased not only the biomass, but also the P concentration of the tomato plants under the low Pi supply condition; however, no significant difference of both the biomass and P concentration could be observed between the colonized and the noncolonized plants under the high Pi supply condition.

**Figure 6:**
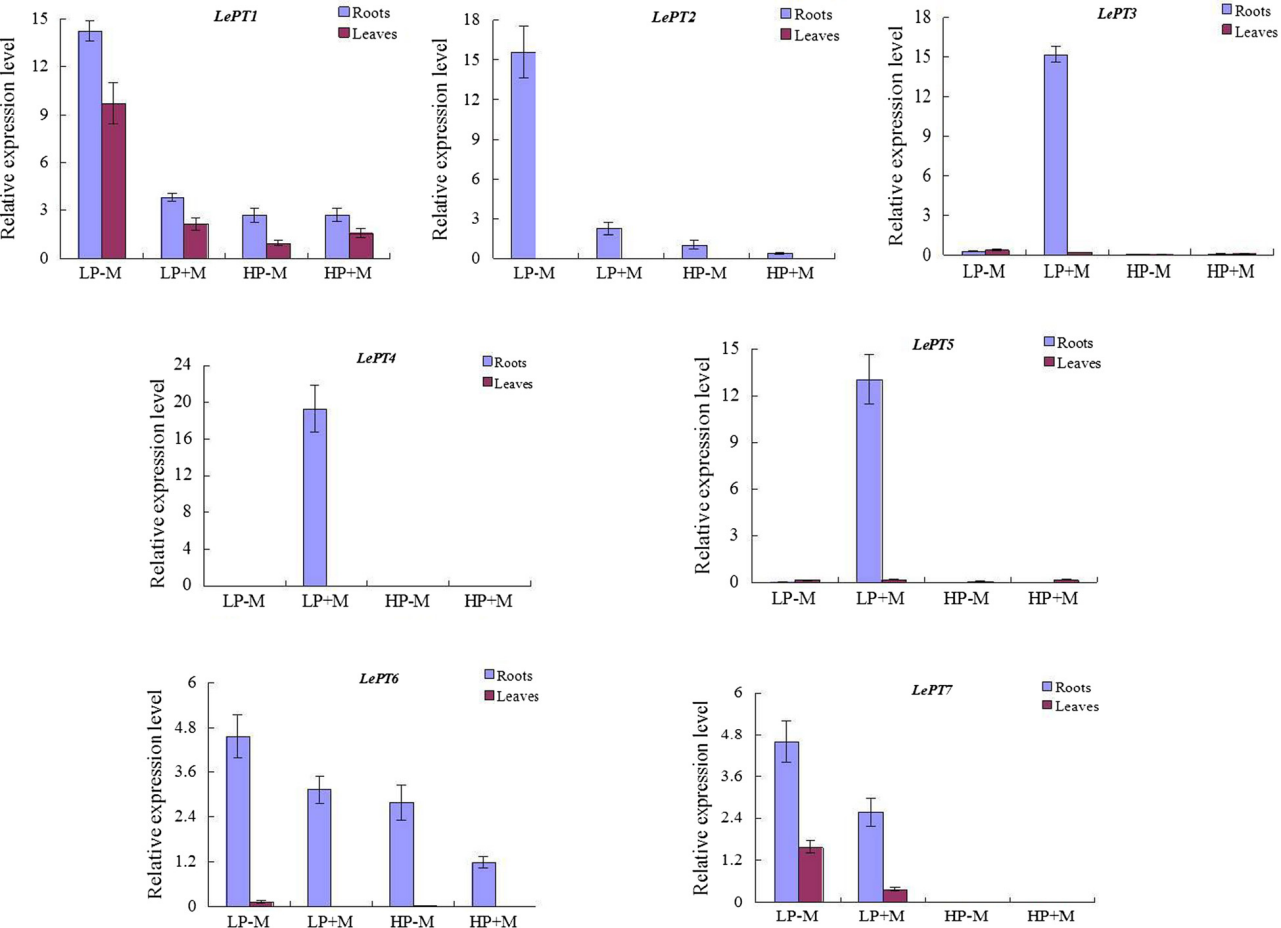
**Real-time RT-PCR analysis of the Pht1 genes in tomato roots and leaves in response to mycorrhizal fungi colonization under high and low Pi supply conditions.** The plants were incubated for two months with a mycorrhizal inoculum containing *Glomus intraradices*. LP and HP represent supply with 0.05 mM and 1 mM Pi, respectively; -M and + M represent the inoculation with autoclaved and active inoculum, respectively. The relative expression levels of each tomato Pht1 gene was also shown as percentage of the constitutive *Actin* expression activity. Each bar was the mean of three biological replications with standard error.

qRT-PCR analysis revealed that except the three paralogues, *LePT3*, *LePT4* and *LePT5*, of which the transcripts were strongly enhanced or specifically activated only in the inoculated roots under the low Pi supply condition, and *LePT8*, of which the transcripts were not detectable in both tissues under any treatments, the expression of the other four paralogues, *LePT1*, *LePT2*, *LePT6* and *LePT7*, were significantly repressed under the high Pi supply condition (Figure 6). Such down-regulated cases occurred more conspicuously upon the two paralogues, *LePT2* and *LePT7*, as their transcripts in both the root and leaf tissues were drastically decreased (*LePT2*) or even completely absent (*LePT7*) under high Pi conditions regardless of with or without AM colonization. In addition, very significant decrease of the transcript abundance of the four paralogues was also detected in both the roots and leaves of the colonized tomato plants as compared to those non-colonized controls under low Pi supply condition (Figure 6). The remarkable down regulation of these four members in response to high-P supply and AMF-colonization might be partially caused by the significant increase of P concentration in such treated tomato plants (Figure 5). Interestingly, although *LePT2* and *LePT6* were considered to be the closest related genes in tomato Pht1 family due to their identical coding sequence, the down regulation of *LePT6* in roots in response to AM symbiosis under low Pi condition was much moderate than that of *LePT2*. Such discrepancy in expression levels strongly suggests that the regulatory components controlling the activation or suppression of *LePT2* and *LePT6* have divergent after the two paralogues produced from a relatively recent duplication event.

**Figure 7:**
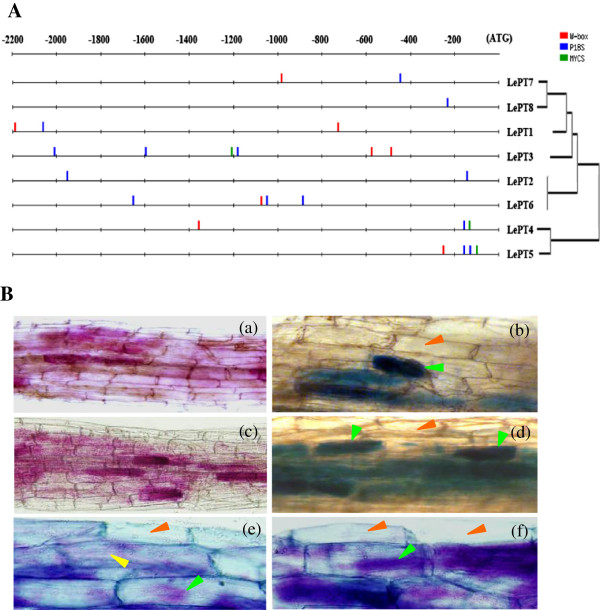
**Analysis of the tomato Pht1 gene promoters. (A)** Comparative analysis of putative *cis*-regulatory elements responsible for the Pi- and AM-regulated expression between the eight tomato Pht1 promoters. Two previously reported Pi-responsive motifs (P1BS and W-box) and one AM-activated motif (MYCS) were searched using the DNA-pattern matching arithmetic (http://rsat.ulb.ac.be/rsat/). P1BS, GNATATNC; MYCS, TTCTTGTTC; W-box, TTGACY. **(B)** Histochemical analysis for the promoter activity of the two AM-induced Pht1 members, *LePT3* and *LePT5*. **(a-d)** Localization of β-glucuronidase (GUS) activity (**a** and **b**, Magenta GUS; **c** and **d**, blue GUS) in mycorrhizal roots driven by the promoters of *LePT3***(a, c)** and *LePT*5 **(b, d)**, respectively. **(e, f)** Co-localization of GUS activity (indicated by the purple color, from the overlay of the Magenta-GUS and Trypan Blue stains) showed that the *LePT3*and *LePT5* promoter fragments (*pLePT3*_*-1250*_ and *pLePT5*_*-471*_) were sufficient to direct GUS expression in mycorrhizal roots and were confined to distinct cortical cells containing AM fungal structures (arbuscules or intracellular hyphae). Green arrows indicate arbuscule or arbusculate hyphae, yellow arrows indicate intracellular hyphae and red arrows indicate noncolonized cells.

The specialized expression profiles of the tomato Pht1 genes in response to AM symbiosis or different Pi status prompted us to investigate their promoter regions. As shown in Figure 7A, the number and localization of the two Pi-regulated (P1BS and W-box) [53,54] and one AM-responsive elements (MYCS) [45,55] differ widely in the promoter regions of these eight Pht1 genes, even though the coding sequence and expression profiles of some paralogues are highly conserved. However, similar as the AM-induced Pht1 genes in other dicot species, the MYCS motif was found to be present exclusively in the putative promoter regions of the three AM-activated Pht1 paralogues, *LePT3*, *LePT4* and *LePT5*, and were located very closely to the Pi-regulated P1BS element [45]. Histochemical staining analysis further revealed that the *LePT3* and *LePT5* promoter regions (*pLePT3*_*-1250*_ and *pLePT5*_*-471*_) containing the two elements, MYCS and P1BS, were sufficient to direct β-glucuronidase (GUS) expression specifically in the mycorrhizal roots and were limited to distinct cells harboring AM fungal structures (arbuscules or intracellular hyphae) (Figure 7B), similar to the cellular distributions of their paralogue LePT4 and other AM-inducible Pht1 homologues from various other plant species reported previously [30,55-57].

## Discussion

In recent studies, benefiting from the availability of whole genome sequence of model plants, dozens of genes belonging to the Pht1 family that encode putative high-affinity Pi transporters have been identified from various plant species using the comparative genome approaches. Tomato, a model species from the Solanaceae family, has been historically characterized to have at least five Pht1 genes [58]. Our present study, through extensive searches of available databases, led to the identification of a total of eight putative Pht1 genes in the tomato genome. As this is the first genome-wide analysis of the Pht1 gene family in any solanaceous species, the investigation of chromosomal organization, evolutionary relationships, as well as expression patterns of the tomato Pht1 genes in this study is of great significance and would offer a basis for better understanding the evolutionary mechanisms underlying the expansion, conservation and functional divergence of the Pht1 genes in the whole Solanaceae family.

### Evolutionary expansion of the tomato Pht1 genes

Multigene families, in a general way, could arise through tandem duplications, resulting in a clustered occurrence, or through genome/segmental duplications, resulting in a discrete distribution of family members [59]. As most of the tomato Pht1 genes were assigned in clusters (such as PT1/PT3, PT2/PT6 and PT4/PT5), with not only very close physical localization (Figure 2), but also very high levels of sequence identity (Table 1, Additional file 5), it is strongly suggestive of that tandem duplications might be the major contributors to the expansion of the tomato Pht1 family. Additionally, since most of the tomato Pht1 members group together with their orthologues from other solanaceous species, such as potato, eggplant and tobacco by forming independent solanaceous clades to the exclusion of other dicot homologues (Figure 3), indicates that the duplications associated with the arising of the coupled paralogues such as *PT1*/*PT3* and *PT4*/*PT5* in solanaceous species, occurred before the speciation of solanaceous lineages from a common ancestor. Intriguingly, in viewing of the localization of tomato *LePT2*/*6* and potato *StPT2*/*6*/*7* on their corresponding chromosomes, it is tempting to make a tendentious conclusion that the duplication giving rise of the tomato *LePT2* and *LePT6* probably occurred before the split of tomato and potato. However, the distribution of the *LePT2* and *LePT6* in the terminal subclades of the phylogenetic tree, and the identical coding sequence shared by them well reflected that the two paralogues were produced from the more recent duplication events that occurred within the tomato lineage postdating it split from a common ancestor shared by potato.

It has been recently documented that the Solanum lineage genome has undergone two rounds of consecutive whole-genome triplication events, one that was ancient and shared with most dicot plant families, and one that was more recent and occurred before the divergence of tomato and potato lineages [34], which led to the hypothesis that segmental duplications produced by genome polyploidy may also exert important impact on the expansion of the Pht1 family. The loci of *LePT2/6* on chromosome 3 and *LePT4/5* on chromosome 6 flanked respectively by two paralogues, *LePht3;3* and *LePht3;4* (with non-homology to the Pht1 genes) (Figure 2) strongly suggests that the arising of the two pairs, PT2/PT6 and PT4/PT5 might originate from a segmental duplication, followed by two independent tandom duplications, which eventually resulted in the fixation of the two couples of Pht1 members on the chromosomes 3 and 6. As the PT4 and PT5 paralogues cluster together with other members from both dicots and monocots in the phylogenetic tree (Figure 3), indicating that the segmental duplication yielding the precursors of the two couples, PT2/PT6 and PT4/PT5, occurred before the divergence of monocots and dicots. With regard to the other four paralogues, the clustered *LePT1* and *LePT3*, and the individual *LePT7* and *LePT8*, they may be the result of several relatively recent segmental or single-gene duplication events that occurred before the speciation of tomato and potato lineages, and followed by at least one independent tandom duplication event (producing the two paralogues, *LePT1* and *LePT3*). It has been well documented that genome polyploidizations are commonly accompanied by massive chromosomal rearrangements [60]. In our study, by comparing the phylogenetic tree and the chromosomal distribution of the tomato and potato Pht1 genes, two segmental inversions leading to the inconsistent linear orders of PT1/PT3 and PT4/PT5 were identified between the two solanaceous genomes, well supporting the very recent findings that at least nine large and several smaller inversions exist between the tomato and potato lineages [41].

### Functional conservation and divergence of the tomato Pht1 gene family

It has been generally accepted that gene duplication followed by functional differentiation has performed a pivotal role in driving evolutionary novelty that allow plants to increase fitness to new environments [47]. To data, as the lack of genome-wide survey of Pht1 genes in any solanaceous species, there is no systematic analysis of tissue-specific expression patterns for the tomato Pht1 family so far. In our present work, we revealed that differential but partial overlapping expression of the Pht1 genes occurred in tomato, as did the members of this family in several other plant species, such as *Arabidopsis*, rice and soybean [13,28,43]. The specialized expression of these genes well mirrors the evolutionary divergence of regulatory elements that are required for controlling Pi uptake and mobilization within/across particular tissues or cells during tomato plant growth.

Earlier results, based on the study of tissue-specific expression and cellular distribution of Pht1 genes in several different plant families revealed that many of the Pht1 genes are expressed dominantly in roots, especially in root epidermis and root hairs, in response to P deprivation, suggesting a potential role of these genes in Pi capture and uptake [12,19]. The transcription data obtained in this study indeed provide direct evidence for strong expression of most of the tomato Pht1 genes in the roots under low Pi supply condition (Figure 6). It was shown that although the transcripts of *LePT1* in the roots and leaves significantly decreased in response to Pi sufficiency, a low level of constitutive expression could be detected throughout the plant, consistent with the expression patterns of its orthologue, *StPT1*, in potato [46], suggesting that *LePT1* and its orthologues may be involved in not only uptake of Pi from soil solution but also redistribution of Pi within plants. *LePT2* has been previously documented to be expressed exclusively in P-depleted roots. However, in the present work, a relatively weak but still observable transcription level could be detected in the roots irrigated with high Pi (1 mM) solution, similar results could also be observed from our previous studies on the *LePT2* orthologues in other three solanaceous species, eggplant, pepper and tobacco [61]. In addition, very slight levels of the *LePT2* transcripts were also detectable in stems, flowers and fruits at green and ripe stages under low Pi supply condition. Although *LePT*2 shares its coding sequence identical to its paralogue, *LePT*6, the transcript abundance of the two members were observably different whether under low Pi supply condition or in response to AM symbiosis. Such discrepancy between the two close paralogues may be caused by the inconsistent distributions of Pi-responsive elements, such as P1BS and W-box, in their promoters (Figure 7A) [53,54,62-64]. Even so, the identical protein activity and high degree of overlapping expression strongly implies the presence of functional redundancy between the two members.

With regard to the three AM-activated Pht1 paralogues, *LePT3*, *LePT4* and *LePT*5, as their transcripts could be induced abundantly only in the inoculated roots under low Pi, but not high Pi condition, similar as the expression of their orthologues from tobacco, pepper, and eggplant, suggesting that the AM-activated expression of *LePT3*-*5*, like their solanaceous orthologues, might also be regulated by at least two conserved *cis*-elements, P1BS and MYCS [45]. Comparative screening of the putative promoter regions of the tomato Pht1 genes indeed led to the identification of the two motifs that were closely and exclusively localized in the promoters of the three AM-activated Pht1 genes (Figure 7A). The relatively high proportion of AM-induced Pht1 genes present/retain in solanaceous species compared to that in many of other mycorrhizal plants, such as rice, to a certain extent, reflects the importance of these genes in regulation of symbiosis during the Solanaceae evolution. In addition, the strong expression levels of these symbiosis-activated Pht1 genes in contrast to the remarkable down-regulation of the Pi transporters responsible for direct Pi uptake in mycorrhizal roots also provided strong evidence to support the earlier findings that symbiotic uptake pathway would contribute the majority of the accumulated Pi received by mycorrhizal tomato and other plants [65-67]. It is worth noting that although *LePT4* was observed to be the gene that had the highest expression level than the other paralogues in tomato mycorrhizal roots under low Pi condition (Figure 6), mutation of the *LePT4* expression in tomato virtually unaffected the establishment of AM symbiosis [30,40], which seems contradictory to the recent findings that knock down/out of the AM-specific or up-regulated Pht1 genes in *Lotus japonicus*, *Medicago* and *Astragalus sinicus* significantly impaired both the development of AM interaction and symbiotic Pi uptake [31,55,68]. The absence of AM-associated phenotypes in the tomato *lept4* knock out mutant was thus suggested to be the genetic redundancy within the tomato Pht1 gene family [30,69]. This explanation might be reasonable as a result of that *LePT4* and its AM-induced paralogue, *LePT5*, were considered to be diverged from a common precursor through tandom duplications (Figure 2), and thus it is unsurprising that *LePT5* would share a similar physiological role with *LePT4* in tomato. Interestingly, although there exist two AM-specific Pht1 genes, the strongly activated *OsPT11* and poorly induced *OsPT13*, in the rice Pht1 family, no functional redundancy was observed between the two paralogues, as silencing whichever of the two paralogues caused a significant repression of AM symbiosis [70]. Since the rice OsPT11 and OsPT13 are distributed relatively distantly in phylogenetic tree, similar as the phylogenetic relationships between solanaceous PT3 and PT4/PT5 paralogues, it is tempting to raise a speculation that no functional redundancy might be existent between the two groups (PT3 and PT4/PT5) of Pht1 transporters in regulation of the development of AM symbiosis.

Interestingly, within some other plant species, such as in soybean, there exists specialized Pht1 member(s) that is (are) mainly expressed in sink tissues, such as in flowers, implying possible functions of these genes in Pi import from source to sink [13,71]. However, in our present study, we did not observe any member in the tomato Pht1 family that is dominantly expressed in the flower or fruit tissue. The relatively high transcription levels of *LePT1* and *LePT7* in these sink tissues compared to the other six paralogues led to the suggestion that the two members, especially the *LePT1*, might have been evolved to meet the requirement of transporting Pi from the source to the sink organs or cells. One of the potential evolutionary fates of gene duplications is considered to silence (nonfunctionalization) one of the duplicate copies [72]. The inactivation of *LePT8* in all the tissues led to the suggestion that *LePT8* might be on the way to become a pseudogene. The presence of several fragments that showed substantial homology to plant Pht1 genes but with incomplete coding regions in the tomato and potato genomes also well supports the theory that genome polyploidizations as well as the following gene loss (diploidization) are common characters of plant genomes [73,74].

## Conclusions

Taken together, this study provided the first comprehensive analysis of the chromosomal organization, phylogenetic evolution and tissue-specific expression patterns for each member of the Pht1 family in tomato. The results presented here could offer a useful basis for future research work on better understanding the mechanisms underlying the evolutionary regulation of Pht1 genes in response to Pi deficiency and AM symbiosis during tomato growth. The high conservation not only in the coding sequence, but also in the chromosomal distribution between the tomato and potato Pht1 orthologues could also lend strong evidence to support the further comparative genomics analysis across the whole Solanaceae family. However, we also realize that although Pht1 family have been commonly considered to be a high-affinity Pi transporter family, an increasing number of members of this family in other plant species have been demonstrated exhibiting a low or even dual affinity for Pi uptake in heterologous yeast or oocyte expression system [75,76]. Therefore, more information, especially the transport kinetics, cellular distributions and physiological phenotypes of knockout/knockdown mutants is needed in the near future to determine more precise functional roles for each of the tomato Pht1 genes.

## Methods

### Plant material and growth conditions

Tomato (*Solanum lycopersicum* cv. *Micro-Tom*), was used in this study. The seeds were surface-sterilized, germinated and maintained in tissue culture for three weeks using 1/2 MS medium supplemented with 1.5% sucrose. The aseptic plantlets were then transferred to pot culture for either with high-/low-P treatment or inoculation with AM fungi.

In pot culture, two plantlets were transplanted to a 3 dm^3^ plastic pot filled with sterilized sand. A sand-based inoculum containing *Glomus intraradices* (*Gi*) was used for inoculation. Each plants was inoculated with 5 g inoculum or autoclaved inoculum around the roots. The plants were grown in a growth room with a 14-h light period (28-30°C) and a 10-h dark period (18-20°C). The irrigating solution contained the following nutrients: 1 mM NH_4_NO_3_, 2 mM KNO_3_, 0.5 mM Ca(NO_3_)_2_, 0.25 mM CaCl_2_, 0.5 mM MgSO_4_, 20 μM Fe-EDTA, 9 μM MnCl_2_, 46 μM H_3_BO_3_, 8 μM ZnSO_4_, 3 μM CuSO_4_, 0.03 μM (NH4)_2_MoO_4_, and 1 mM (high-P treatment) or 0.05 mM Pi (low-P treatment) NaH_2_PO_4_. The experiment comprised 4 replicates for each treatment. After inoculation for six weeks, the plants were either harvested for collecting the root, stem and young leaf samples or for continuing growing for the later collection of flower and fruit samples at young and ripe stages. The collected samples were immediately frozen in liquid nitrogen and stored at -80°C for subsequent RNA isolation.

### Identification of Pht1 genes in tomato and potato genomes

Members of Pht1 gene family in the tomato genome were identified using the BLASTN and TBLASTN algorithm wrapped in the BLAST 2.2.27+ applications. To identify the potential Pht1 genes in tomato genome, coding sequence (cds) and deduced protein sequences of the *Arabidopsis* and rice Pht1 genes were queried respectively in the tomato genomic sequence database downloaded from the Solanaceae Genomics Network (http://www.sgn.cornell.edu). Sequences with a query over 50% and e-value less than -10 were taken as the Pht1 candidates. All the obtained sequences were submitted to NCBI (http://www.ncbi.nlm.nih.gov/) and Pfam database (http://www.sanger.ac.uk/Software/Pfam/search.shtml) for further confirmative analysis. For chromosomal localization analysis, the tomato Pht1 candidates were further used as queries for BLASTN searches against the SGN Tomato Whole Genome Scaffolds data (2.40) (http://solgenomics.net/organism/Solanum_lycopersicum/genome).

For identification of the potential homologues of tomato Pht1 genes from potato genome, the potato genome sequence data downloaded from the SGN database (http://solgenomics.net/organism/Solanum_tuberosum/genome) were also extensively searched using the tomato Pht1 genes as queries. The naming of the potato Pht1 genes were partially based on their phylogenetic relationships with the tomato homologues.

### Phylogenetic analysis

The sequence data used in this study were collected using a query search in the NCBI database using the known Pht1 family gene sequences from *Arabidopsis* and rice. Multiple sequence alignments were performed using the program ClustalX (version 1.8) with default gap penalties. An un-rooted phylogenetic tree was generated using the deduced amino acid sequences of Pht1 genes by neighbor-joining algorithms wrapped in the MEGA 5.1 phylogeny program (http://www.megasoftware.net). Bootstrap analysis was carried out with 1,000 replicates.

### RNA extraction and real-time RT-PCR analysis

Total RNA was isolated from 100 mg of various tissue samples, including roots, stems, young leaves and flowers, using the guanidine thiocyanate extraction method with Trizol reagent (Invitrogen) and from fruit samples using CTAB-sour phenol extraction method as described by Chang [77]. After extraction, the RNA samples were treated with DNase I (TaKaRa) to eliminate the trace contaminants of genomic DNA. For conducting reverse transcription (RT) PCR analysis, approximately two micrograms of total RNA from each sample was used to synthesize first-strand cDNA using a reverse transcription kit (TaKaRa), and the synthesized cDNAs were used as templates in the following PCR reactions.

Real-time RT-PCR analysis was performed to relatively quantify the expression levels of tomato Pht1 genes in different tissues or in roots and leaves in response to mycorrhizal colonization at high and low P status. The reaction was conducted on the Applied Biosystems (ABI) Plus Real-Time PCR System using the SYBER premix ExTaq kit (TaKaRa). Relative quantification of the transcripts for each tomato Pht1 gene was standardized to the expression level of the tomato constitutive *Actin* gene, calculated by the formula Y = 10^-(ΔCt/3)^ × 100% (ΔCt is the differences of cycle threshold value between the target Pht1 gene and the control *Actin* products) [47,78]. The specificity of primer sets designed for the qRT-PCR (Additional file 6) was confirmed by sequencing after the PCR reaction.

### Histochemical GUS staining and detection of mycorrhizal fungal colonization

A 1250-bp *LePT3* promoter fragment and a 471-bp *LePT5* promoter fragment immediately upstream of the translation initiator ATG were amplified and cloned into binary vector pBI12, respectively, to replace the CaMV35S promoter in front of the β-glucuronidase (GUS) gene. The resulting two constructs were designated as *pLePT3*_*-1250*_ and *pLePT5*_*-471*_, respectively, and introduced into *Agrobacterium tumefaciens* strain EHA105 for genetic transformation.

Histochemical GUS staining of the fresh transgenic roots was performed as described previously [44]. For visualization of fungal structures, the Magenta-GUS stained root segments were treated with 10% KOH solution heated to 90°C for 1 h, and then neutralized with 1% HCl (v/v) solution for 5 min. The root materials were then counter-stained with 0.3% trypan blue solution for 2 h at 90°C. The co-localization of Magenta-GUS and the trypan blue staining were indicated by purple color. The stained materials were rinsed in 50% glycerol and photographed by a stereomicroscope with a color CCD camera (Olympus).

## Abbreviations

PT: Phosphate transporter; EST: Expressed sequence tag; AM: Arbuscular mycorrhizal; AMF: Arbuscular mycorrhizal fungi; Indels: Insertions and deletions.

## Competing interests

The authors declare that they have no competing interests.

## Authors’ contributions

AQC and GHX contributed to the experimental design and manuscript drafting. MG contributed to the manuscript editing. XC, HMW and DHL performed the RNA extraction, primer design, RT-PCR validation. AQC and HYQ performed the bioinformatics analysis. All authors have read and approved the final manuscript.

## Supplementary Material

Additional file 1 Pht1 members identified in tomato genome. Click here for file

Additional file 2 **Alignment of the coding sequences and putative untranslated regions of *LePT2* and *LePT6*.** Click here for file

Additional file 3 **Alignment of the partial coding sequences of *LePT7* and the pseudogene *LePTx*.** Click here for file

Additional file 4 Pht1 genes identified in potato genome. Click here for file

Additional file 5 Comparison of the amino acid sequences of Pht1 homologous genes from tomato and potato. Click here for file

Additional file 6 Gene-specific primers used for Real-time RT-PCR amplification of tomato Pht1 genes. Click here for file
