# Supplementary material for: Genome-wide investigation and expression analysis suggest diverse roles and genetic redundancy of Pht1 family genes in response to Pi deficiency in tomato
Source: BMC Plant Biol. 2014 Mar 11;14:61. doi: 10.1186/1471-2229-14-61 (PMC4007770; doi:10.1186/1471-2229-14-61)
Supplement: Additional file 1 — Pht1 members identified in tomato genome. [file 1471-2229-14-61-S1.doc]

**Additional file 1. Pht1 members identified in tomato genome**

| **Gene** | **Chr** | **Start** | **End** | **ORF length** | **Protein**  **length** | **Mol Wt**  **(KDa)** | **p*I*** | **Intron** | **P.L** | **TMs** |
| --- | --- | --- | --- | --- | --- | --- | --- | --- | --- | --- |
| ***LePT1*** | 9 | 64973106 | 64974722 | 1617 | 538 | 58.71 | 8.43 | 0 | PM | 12 |
| ***LePT2*** | 3 | 400886 | 399300 | 1587 | 528 | 57.77 | 8.50 | 0 | PM | 12 |
| ***LePT3*** | 9 | 64981619 | 64983223 | 1605 | 534 | 58.54 | 8.68 | 0 | PM | 12 |
| ***LePT4*** | 6 | 32057715 | 32056126 | 1590 | 529 | 58.69 | 8.04 | 0 | PM | 12 |
| ***LePT5*** | 6 | 32060987 | 32059398 | 1590 | 529 | 59.04 | 8.73 | 0 | PM | 12 |
| ***LePT6*** | 3 | 412992 | 414578 | 1587 | 528 | 57.77 | 8.50 | 0 | PM | 12 |
| ***LePT7*** | 9 | 60426552 | 60428153 | 1602 | 533 | 58.31 | 8.92 | 0 | PM | 12 |
| ***LePT8*** | 6 | 20519555 | 20521159 | 1605 | 534 | 58.49 | 8.92 | 0 | PM | 12 |

Chr: chromosome; Mol Wt: molecular weight; p*I*: protein isoelectric point; P.L: predicted protein localization; PM: plasma membrane; TMs: trans-membrane domains.
