# Supplementary material for: Genome-wide investigation and expression analysis suggest diverse roles and genetic redundancy of Pht1 family genes in response to Pi deficiency in tomato
Source: BMC Plant Biol. 2014 Mar 11;14:61. doi: 10.1186/1471-2229-14-61 (PMC4007770; doi:10.1186/1471-2229-14-61)

**Additional file 2.** The alignment of the coding sequences and putative untranslated regions (3’ and 5’ UTRs) of *LePT2* and *LePT6*. The start codon (ATG) and stop codon (TGA) are indicated by arrows.


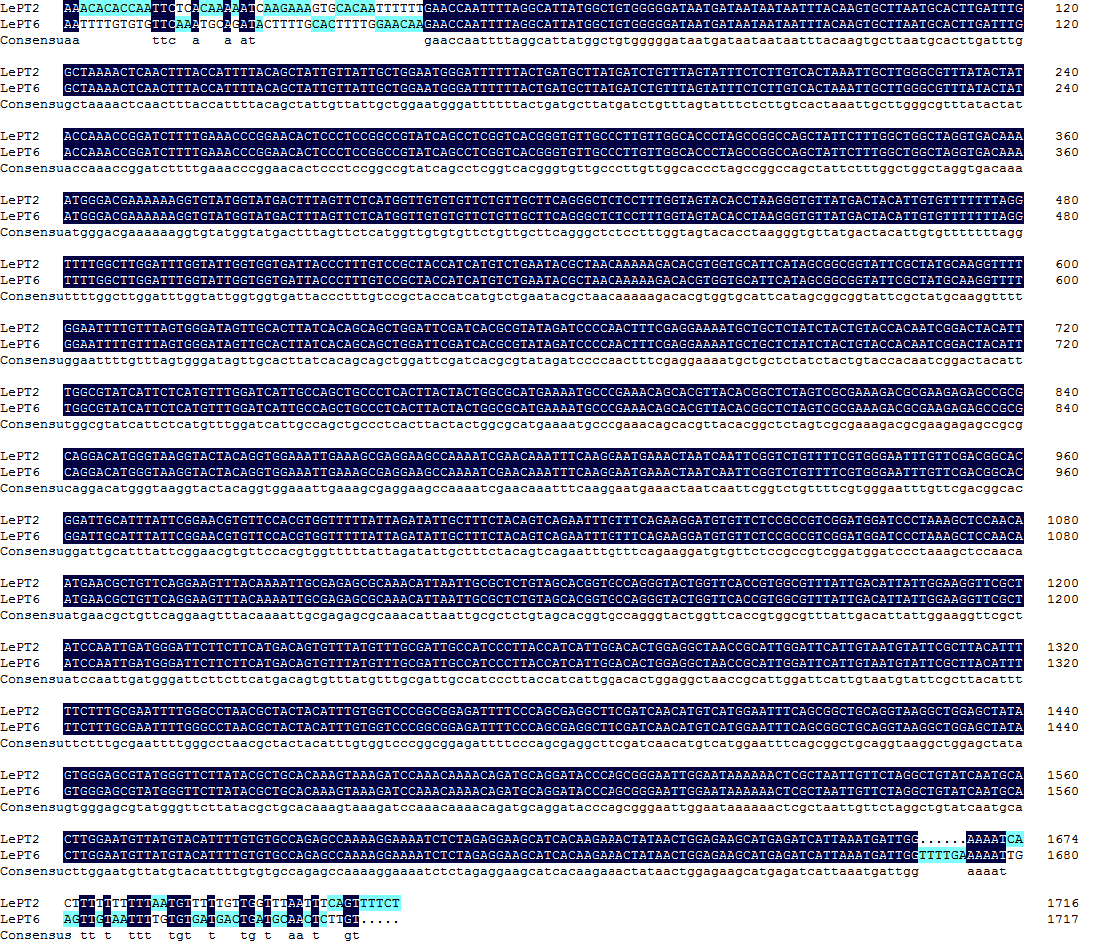

Supplement: Additional file 2 — Alignment of the coding sequences and putative untranslated regions of LePT2 and LePT6 . [file 1471-2229-14-61-S2.doc]
