# Supplementary material for: Genome-wide investigation and expression analysis suggest diverse roles and genetic redundancy of Pht1 family genes in response to Pi deficiency in tomato
Source: BMC Plant Biol. 2014 Mar 11;14:61. doi: 10.1186/1471-2229-14-61 (PMC4007770; doi:10.1186/1471-2229-14-61)

**Additional file 3.** The alignment of the partial coding sequences of *LePT7* and *LePTx*. The stop codons (TAG, TAA and TAG) caused by nonsense mutations or frame-shift indels (insertions and deletions) (indicated by arrows) within the putative coding regions of *LePTx* were boxed.


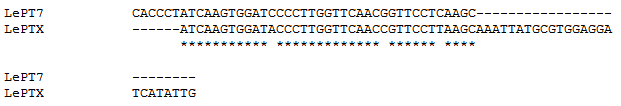

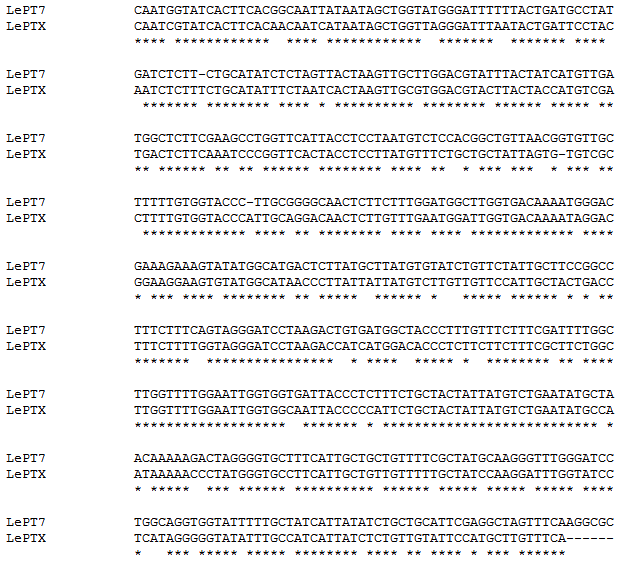

Supplement: Additional file 3 — Alignment of the partial coding sequences of LePT7 and the pseudogene LePTx . [file 1471-2229-14-61-S3.doc]
