# Supplementary material for: Genome-wide investigation and expression analysis suggest diverse roles and genetic redundancy of Pht1 family genes in response to Pi deficiency in tomato
Source: BMC Plant Biol. 2014 Mar 11;14:61. doi: 10.1186/1471-2229-14-61 (PMC4007770; doi:10.1186/1471-2229-14-61)
Supplement: Additional file 4 — Pht1 genes identified in potato genome. [file 1471-2229-14-61-S4.doc]

**Additional file 4. Pht1 genes identified in potato genome**

| **Gene** | **Chr.** | **Start** | **End** | **ORF length** | **Protein**  **length** | **Mol. Wt.**  **(KDa)** | **p*I*** | **Intron** | **P.L** | **TMs** |
| --- | --- | --- | --- | --- | --- | --- | --- | --- | --- | --- |
| *StPT1* | 9 | 48156747 | 48155131 | 1617 | 538 | 58.78 | 8.75 | 0 | PM | 12 |
| *StPT2* | 3 | 826074 | 824494 | 1581 | 526 | 57.63 | 8.73 | 0 | PM | 12 |
| *StPT3* | 9 | 48151091 | 48149484 | 1608 | 535 | 58.57 | 8.84 | 0 | PM | 12 |
| *StPT4* | 6 | 33884774 | 33886363 | 1590 | 529 | 58.73 | 8.73 | 0 | PM | 12 |
| *StPT5* | 6 | 33881410 | 33882999 | 1590 | 529 | 59.02 | 8.87 | 0 | PM | 12 |
| *StPT6* | 3 | 835653 | 834076 | 1578 | 525 | 57.52 | 8.74 | 0 | PM | 12 |
| *StPT7* | 3 | 842698 | 844275 | 1578 | 525 | 57.46 | 8.74 | 0 | PM | 12 |
| *StPT8* | 9 | 39451575 | 39453182 | 1608 | 535 | 58.71 | 9.19 | 0 | PM | 11 |
| *StPT9* | 9 | 42962230 | 42959853 | 1593 | 530 | 58.05 | 8.13 | 2 | PM | 11 |
| *StPT10* | 9 | 42879795 | 42881279 | 1485 | 494 | 53.84 | 8.20 | 0 | PM | 11 |

Chr: chromosome; Mol Wt: molecular weight; p*I*: protein isoelectric point; P.L: protein localization; PM: plasma membrane; TMs: trans-membrane domains.
