# Supplementary material for: Genome-wide investigation and expression analysis suggest diverse roles and genetic redundancy of Pht1 family genes in response to Pi deficiency in tomato
Source: BMC Plant Biol. 2014 Mar 11;14:61. doi: 10.1186/1471-2229-14-61 (PMC4007770; doi:10.1186/1471-2229-14-61)
Supplement: Additional file 5 — Comparison of the amino acid sequences of Pht1 homologous genes from tomato and potato. [file 1471-2229-14-61-S5.doc]

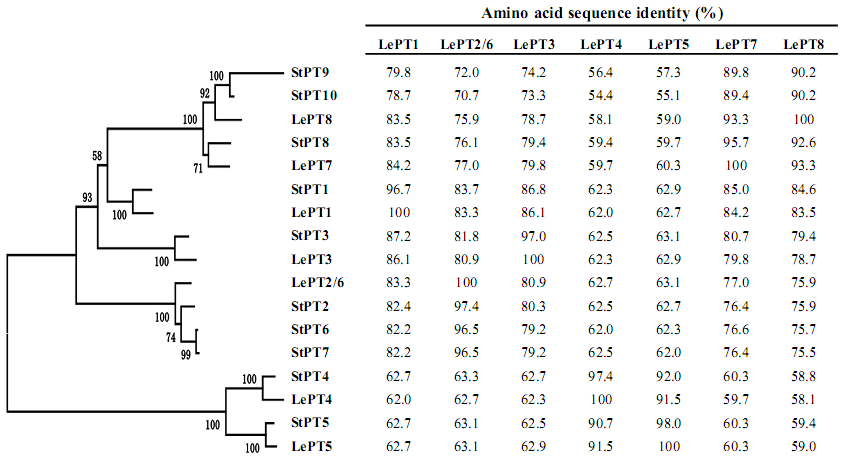
**Additional file 5.** Comparison of the amino acid sequences of Pht1 homologous genes from tomato and potato. Sequences are ordered according to the phylogenetic relationship of the corresponding protein sequences, as shown on the left. Identity matrix for the deduced amino acid sequences of tomato and potato Pht1 homologues were shown on the right.
