# Supplementary material for: Genome-wide investigation and expression analysis suggest diverse roles and genetic redundancy of Pht1 family genes in response to Pi deficiency in tomato
Source: BMC Plant Biol. 2014 Mar 11;14:61. doi: 10.1186/1471-2229-14-61 (PMC4007770; doi:10.1186/1471-2229-14-61)
Supplement: Additional file 6 — Gene-specific primers used for Real-time RT-PCR amplification of tomato Pht1 genes. [file 1471-2229-14-61-S6.doc]

**Additional file 6.** Gene-specific primers used for Real-time RT-PCR amplification of tomato Pht1 genes.

| Gene | Forward Primer | Reverse Primer |
| --- | --- | --- |
| *LePT1* | aggggaagaggaaactgtagctg | ataccacaaattaactcaaactgcat |
| *LePT2* | gccagagccaaaaggaaaatc | tgcaacaaacaagcttacacaataca |
| *LePT3* | ttgtgttaggttgtgtgaattttctt | agctctttgcacgatcttaaatgac |
| *LePT4* | cgggcagaatgagacacagatg | tgaagatagaaagcacaaggcgtagt |
| *LePT5* | gcagaacgagacgcagatgaa | tgctgaatttgataaacttgccaa |
| *LePT6* | gccagagccaaaaggaaaatc | aagagttgcatcagtcatcacaca |
| *LePT7* | aggggaagtcactggaagagatg | gcagcaatgacagataacctaatacgt |
| *LePT8* | aagggaaaacgaagactcagcac | aggttgaggtaaagaaactatagtgct |
| *Actin* | ttccgttgcccagaggtcct | tcgccctttgaaatccacatc |
